# Parents’ Perspectives on Using Artificial Intelligence to Reduce Technology Interference During Early Childhood: Cross-sectional Online Survey

**DOI:** 10.2196/19461

**Published:** 2021-03-15

**Authors:** Jill Glassman, Kathryn Humphreys, Serena Yeung, Michelle Smith, Adam Jauregui, Arnold Milstein, Lee Sanders

**Affiliations:** 1 Clinical Excellence Research Center School of Medicine Stanford University Stanford, CA United States; 2 Department of Psychology and Human Development Vanderbilt University Nashville, TN United States; 3 Department of Biomedical Data Science School of Medicine Stanford University Stanford, CA United States; 4 Division of General Pediatrics School of Medicine Stanford University Stanford, CA United States

**Keywords:** parenting, digital technology, mobile phone, child development, artificial intelligence

## Abstract

**Background:**

Parents’ use of mobile technologies may interfere with important parent-child interactions that are critical to healthy child development. This phenomenon is known as technoference. However, little is known about the population-wide awareness of this problem and the acceptability of artificial intelligence (AI)–based tools that help with mitigating technoference.

**Objective:**

This study aims to assess parents’ awareness of technoference and its harms, the acceptability of AI tools for mitigating technoference, and how each of these constructs vary across sociodemographic factors.

**Methods:**

We administered a web-based survey to a nationally representative sample of parents of children aged ≤5 years. Parents’ perceptions that their own technology use had risen to potentially problematic levels in general, their perceptions of their own parenting technoference, and the degree to which they found AI tools for mitigating technoference acceptable were assessed by using adaptations of previously validated scales. Multiple regression and mediation analyses were used to assess the relationships between these scales and each of the 6 sociodemographic factors (parent age, sex, language, ethnicity, educational attainment, and family income).

**Results:**

Of the 305 respondents, 280 provided data that met the established standards for analysis. Parents reported that a mean of 3.03 devices (SD 2.07) interfered daily in their interactions with their child. Almost two-thirds of the parents agreed with the statements “I am worried about the impact of my mobile electronic device use on my child” and “Using a computer-assisted coach while caring for my child would help me notice more quickly when my device use is interfering with my caregiving” (187/281, 66.5% and 184/282, 65.1%, respectively). Younger age, Hispanic ethnicity, and Spanish language spoken at home were associated with increased technoference awareness. Compared to parents’ perceived technoference and sociodemographic factors, parents’ perceptions of their own problematic technology use was the factor that was most associated with the acceptance of AI tools.

**Conclusions:**

Parents reported high levels of mobile device use and technoference around their youngest children. Most parents across a wide sociodemographic spectrum, especially younger parents, found the use of AI tools to help mitigate technoference during parent-child daily interaction acceptable and useful.

## Introduction

### Technology Interference in Responsive Parenting

Parent and caregiver behaviors that foster healthy, nurturing environments during early childhood produce significant, cost-effective benefits across the life course well into adulthood [1-3]. Exposure to such environments during the infant and toddler years—including verbally rich interactions, affective-emotional caregiver behaviors (eg, returning the baby’s gaze, positive touch), and the maintenance of the child’s focus of interest—is associated with positive cognitive, linguistic, physical, and socioemotional health outcomes [4-6]. However, over the past decade, a new threat to these environments has emerged: the use of mobile technologies by parents during active caregiving [7-9]. In fact, recent studies suggest that this parenting technoference is associated with reduced adult responsiveness and early child behavior problems [8-10]. Furthermore, these risks to subsequent lifelong health may be highest in low-income households, which disproportionately rely on mobile technology for access to the web and social support [11].

### Early Childhood Parenting Interventions

Interventions most effective in supporting long-term outcomes for children focus on helping parents foster nurturing environments during early childhood [12-15]. Many of these interventions include technological support for wider dissemination or the use of digital technologies as a mode of implementation.For example, the use of audio-detection technology to improve the quality of early childhood literacy environments is used by the Language ENvironment Analysis (LENA) word count device to monitor the linguistic development of young children and provide feedback to parents [16-19]. In another example, the Video Interaction Project provides live coaching feedback to parents based on 5-minute videos of parent-child interactions during regularly scheduled well-child visits [20,21]. However, the moderating effects of such parenting interventions on technoference have not yet been studied. Furthermore, human, financial, and technological constraints limit their scalability.

### Augmenting Parenting Interventions With Artificial Intelligence–Based Tools

Emergent artificial intelligence (AI) capabilities—including noninvasive visual and audio monitoring tools that use trained algorithms to automatically detect certain human behaviors—may enable more efficient and scalable behavioral observation to augment interventions that support key nurturing parenting behaviors. In fact, in both research and commercial applications, this has proven to be successful; wearable devices support adult lifestyle changes (eg, physical activity), and more recently in hospital settings, computer vision is being employed to promote adherence to patient care protocols such as hand hygiene and patient mobilization [22,23]. Given the increasing presence of mobile devices in the parent-child environment, AI-based technology may be an important tool for identifying and implementing interventions that help parents reduce technology interference in their relationships with their young children. However, little is known about the acceptability of using AI tools to further identify and mitigate the effects of parent technoference.

### Acceptability of New Technology

One of the most common models used to understand and measure the acceptability of new technology in general, and in health care in particular, is the technology acceptance model (TAM). Originally developed in the context of the adoption of workplace technology tools, the TAM posits that the perceived usefulness (PU) and the ease of use of a new technology determine its acceptability, which in turn influences the intention to use and actual use of the new technology [24-26]. In addition, the literature on the acceptability of health- and behavior-related technology, such as wearables, telemedicine, and mobile health apps, emphasizes that individual sociodemographic factors such as age, gender, and income also play important roles and should be considered in any early technology-based intervention development processes [27-30]. More specifically, there is some evidence that parental engagement in technology-assisted parenting interventions may be higher for younger parents, with mixed findings on whether lower- or higher-income parents are more likely to use these interventions [14].

A review of parental engagement in psychological interventions for their children indicated that beliefs about the severity of the problem may influence engagement with the treatment [31]. Furthermore, the widely used Health Belief Model (HBM) suggests that parents who are more aware of their susceptibility to the problem of technoference would be more accepting of solutions for addressing it [32]. Similar to the TAM, the HBM also suggests that the more useful parents perceive AI-based tools, the more likely they will be to adopt them. However, these theories have not yet been tested in the emerging context of technoference and AI-based tools to reduce it.

### Study Goals

Given these gaps, the overall purpose of this exploratory study is to investigate the magnitude and potential determinants of the acceptability of AI-based tools for assisting parents in reducing parenting technoference. In particular, the goals were to assess (1) parental perceptions of their own technoference and its harms, (2) the acceptability of AI-based tools to reduce the negative effects of technoference, (3) the variation in acceptability and technoference across sociodemographic factors, and (4) the relative importance of parents’ perceptions of their own technoference and sociodemographic factors in relation to acceptability.

## Methods

### Design

We conducted a cross-sectional observational study using a population-based survey on the web of US parents of children aged ≤5 years. The survey was developed for the Amazon TurkPrime (now called CloudResearch) platform for recruitment and administration through the Prime Panels service. Prime Panels is a research participant recruitment platform developed as a step up from Amazon Mechanical Turk (MTurk), in that it includes a set of validated attention and language comprehension screening questions [33,34]. The survey was pilot tested for comprehension on a convenience sample of 4 mothers from the San Francisco Bay Area. The survey was then translated into the Spanish language by a trained, bilingual research associate and back-translated from Spanish to English by another independent, trained bilingual research associate; differences between the original and back-translated versions were resolved in Spanish by a third bilingual research coordinator. Participants could choose to complete the survey in Spanish or English. Recruitment began on May 24, 2019, and ended on June 13, 2019. All procedures were approved by the Stanford Human Subjects Research Office (Institutional Review Board protocol number 50428).

### Participants

Eligibility criteria were adults aged >18 years, with primary caregiving responsibility for at least one child aged <5 years in the household. Exclusion criteria included the inability to read English or Spanish or completion of the survey outside the United States. Using the Prime Panels sampling frame, we aimed to recruit a sample representative of the US population, with >25% respondents self-reporting an underrepresented minority status (Hispanic, Black, and Asian or Pacific Islander), >25% with educational attainment less than a college degree, and >15% monolingual Spanish speakers.

### Survey Items

The survey consisted of items adapted from existing validated surveys covering the following domains: parenting and child behavior priorities, parents’ perceptions of their technology use, parents’ perceptions of their technology use in the presence of their child (technoference), the acceptability of AI-based tools to help reduce parenting technoference, and sociodemographic factors. The full survey can be found in Multimedia Appendix 1.

#### Primary Outcome

The primary outcome was technology acceptance, defined as the acceptability and perceived utility of an AI-based tool for reducing parenting technoference and measured by averaging responses to the following items having 6-point Likert scale response options (strongly disagree to strongly agree): (1) “Using a computer-assisted coach while caring for my child would help me be more aware of my device use around my child,” (2) “Using a computer-assisted coach while caring for my child would improve my interactions with my child,” (3) “Using a computer-assisted coach while caring for my child would help me be a better parent,” (4) “Using a computer-assisted coach while caring for my child would help me notice more quickly when my device use is interfering with my caregiving,” (5) “Using a computer-assisted coach while caring for my child would help me keep my attention focused on my child,” and (6) “Using a computer-assisted coach while caring for my child would be useful to me.” The items were preceded by the following statement:

Some electronic devices are currently being designed to HELP you have a better connection with your child—by coaching you or giving you meaningful, real-time feedback. Imagine such a “computer-assisted coach,” which you could use in your home to get feedback on your use of electronic devices while caring for your child. The computer-assisted coach would automatically analyze computer vision and other data to provide the feedback. Whenever you want, you could turn this computer-assisted coach on or off.

These items were adapted from the PU scale of the TAM (TAM-PU) [24-26,35].

#### Secondary Outcomes and Independent Variables

Problem technology use was defined as the extent to which parents perceived that their mobile technology use had risen to a problematic level and was measured using a previously validated scale [9], which averaged responses to the following three items: “When my mobile electronic device alerts me to indicate new messages, I cannot resist checking them”; “I often think about calls or messages I might receive on my mobile phone”; and “I feel like I use my mobile phone too much.” The response options for all three items were on a 6-point scale ranging from strongly disagree to strongly agree. Finally, perception of parenting technoference was measured based on a previously validated index [9], which summed the count of dichotomized responses to the following questions across each of the 6 device types (television, computer, smartphone, tablet, other handheld devices [eg, iPod], and video game device): “In a typical day, how many times does each of the following devices interrupt a conversation or activity between you and your child?” Possible responses in our survey used a 5-point scale (never, 1 time, 2 times, 3 times, and 4 or more times) and were dichotomized to 0 times versus 1 or more times.

Sociodemographic characteristics included self-report of age, sex, race or ethnicity, language spoken at home (English or Spanish), marital status, number of children, education level, working status, and income level. A proxy measure of the geographic region in which the participant resided was derived from the longitude and latitude values of the survey respondent’s computer captured by Amazon TurkPrime. The geographic region was categorized into the 4 US census regions defined by the West, South, Northeast, and Midwest.

### Data Analysis

Survey responses were first analyzed to assess their distribution. Data quality assessments were conducted to identify speeders (those who answer unreasonably fast) and straightliners (those who answer with identical values for each survey item in a block). We defined speeders as anyone who finished the survey at an average speed of less than 2 seconds per question, and we defined straightliners as anyone who had a standardized scale point variation value of −3.79 [36,37]. The internal consistency of the problem technology use and technology acceptance scale was assessed using the Cronbach α. The Cronbach α was not calculated for parenting technoference*,* given that it is a sum of items not necessarily expected to be related [9].

Descriptive statistics were used to address goals 1 and 2, and bivariate analyses were conducted to address goal 3. Given that goal 4 was simply to explore relative, independent associations between independent variables represented by problem technology use, parenting technoference, and sociodemographic factors and the dependent variable technology acceptance rather than to determine multiple causal pathways, we selected multiple regression analysis as the most straightforward, parsimonious way to accomplish this goal [38]. A screening criterion of *P*<.15 was used to determine the sociodemographic characteristics sufficiently associated with the technology acceptance outcome to be entered into the multiple regression models. This *P* value was selected for the purposes of screening as it has been shown that model-building strategies using a .05 criterion often result in the omission of covariates known to be important [39,40].

Multiple linear regression was conducted on full (all sociodemographic covariates) and reduced (only covariates meeting the screening criteria) models. Effect size was estimated using Cohen *d* values or odds ratios (continuous and categorical independent variables) and η^2^ values, representing the proportion of total variance in the technology acceptance outcome explained by each independent variable [41].

Mediation analysis was conducted to further investigate the relative importance of the roles played by problem technology use in general and the more specific parenting technoference measure in influencing technology acceptance. The purpose of the mediation analysis was to help assess whether to focus potential behavior change levers on perceived excessive technology use in general and/or technology interference in parenting specifically. All analyses were conducted using the R version 3.5.3 (R Core Team).

## Results

### Sample

The initial sample of consenting participants meeting the inclusion criteria consisted of 305 survey respondents. Data quality analyses to identify and remove speeders and straightliners resulted in a final analytic sample of 280 observations.

The mean age of the respondents in the final analytic sample was 33 (SD 8) years. In total, 79.2% (222/280) of respondents were female (Table 1), 14.8% (14/270) self-identified as Hispanic, 8.9% (24/270) self-identified as Black, and 5.9% (16/270) self-identified as Asian. Approximately one-third (81/280, 28.9%) of the participants reported less than a high school education, and 13.9% (39/280) spoke a language other than English at home.

**Table 1:** Descriptive statistics for the study sample (N=280).

Characteristics		Values^a,b^
Age (years), mean (SD)		33 (8)
**Gender, n (%)**		
	Female	222 (79.2)
	Male	57 (20.4)
	Other	1 (0.4)
**Race, n (%)**		
	White	184 (68.1)
	Black	24 (8.9)
	Hispanic	40 (14.8)
	Asian	16 (5.9)
	Other	6 (2.2)
**Language spoken at home, n (%)**		
	English	241 (86.1)
	Other	39 (13.9)
**Children, n (%)**		
	1	108 (38.6)
	>1	172 (61.4)
**Education, n (%)**		
	Less than high school	81 (28.9)
	Some college	65 (23.2)
	Higher than or equal to a college degree	134 (47.9)
**Income (US $), n (%)**		
	<25,000	57 (20.4)
	25,000-49,999	68 (24.3)
	50,000-74,999	65 (23.2)
	75,000-100,000	49 (17.5)
	>100,000	41 (14.6)
**Geographic area, n (%)**		
	Midwest	57 (20.4)
	Northeast	49 (17.5)
	South	117 (41.8)
	West	57 (20.4)

^a^Total sample size differs across characteristics due to missing values.

^b^Some percentages add up to slightly less than 100% because of missing values.

### Descriptive Statistics and Bivariate Associations

#### Technology Acceptance

Approximately two-thirds of the respondents (184/282, 65.2% and 175/283, 61.8%, respectively) agreed with the statements “Using a computer-assisted coach while caring for my child would help me notice more quickly when my device use is interfering with my caregiving” and “Using a computer-assisted coach while caring for my child would help me be more aware of my device use around my child.” The internal consistency of the technology acceptance scales was high, with a Cronbach α of .94 (Table 2). The mean level for the technology acceptance outcome was 3.53 (SD 1.29) on a 6-point scale, where higher values represent higher levels of acceptance.

**Table 2:** Bivariate associations between sociodemographic characteristics and technology acceptance, problem technology use, and parenting technoference.

Measure			Problem technology use	*P* value	Parenting technoference	*P* value	Technology acceptance	*P* value
Cronbach α			.80	N/A^a^	N/A	N/A	.94	N/A
Overall, mean (SD)			3.72 (1.32)	N/A	3.03 (2.07)	N/A	3.53 (1.29)	N/A
Age of parent (years; ρ)			−0.12	.04^b,c^	−0.12	.05	−0.11	.07
**Sex, mean (SD)**				.20		.99		.04^c^
	Female		3.67 (1.28)		3.03 (2.03)		3.46 (1.25)	
	Male		3.89 (1.51)		3.05 (2.25)		3.79 (1.42)	
**Education, mean (SD)**				.17		.13		.11
	Less than high school		3.51 (1.39)		3.23 (2.12)		3.34 (1.45)	
	Some college		3.68 (1.22)		2.58 (2.07)		3.42 (1.12)	
	Higher than or equal to a college degree		3.86 (1.33)		3.11 (2.04)		3.69 (1.25)	
**Race or ethnicity, mean (SD)**				.03^c^		.02^c^		.23
	White non-Hispanic		3.63 (1.36)		2.82 (2.0)		3.43 (1.34)	
	White Hispanic		3.67 (1.32)		4.0 (2.11)		3.78 (1.25)	
	Black		4.14 (1.02)		2.96 (2.16)		3.72 (1.13)	
	Other^d^		4.32 (1.21)		3.23 (2.18)		3.89 (1.16)	
**Language at home, mean (SD)**				.50		.005^c^		.92
	English		3.69 (1.33)		2.88 (2.03)		3.52 (1.3)	
	Other		3.88 (1.27)		3.92 (2.16)		3.6 (1.2)	
**Income (US $), mean (SD)**				.11		.91		.14
		<25,000	3.93 (1.08)		2.91 (1.98)		3.54 (1.29)	
		25,000-<49,999	3.45 (1.38)		2.96 (2.28)		3.24 (1.15)	
		50,000-<74,999	3.64 (1.42)		3.2 (2.01)		3.5 (1.39)	
		75,000-<100,000	3.64 (1.12)		2.98 (2.11)		3.79 (1.15)	
		≥100,000	4.06 (1.54)		3.07 (1.97)		3.7 (1.44)	
**Children at home, mean (SD)**				.75		.90		.23
		1	3.66 (1.41)		3.06 (2.1)		3.64 (1.24)	
		>1	3.75 (1.27)		3.01 (2.06)		3.46 (1.31)	
**Geographic region, mean (SD)**				.78		.76		.96
		Midwest	3.76 (1.19)		2.91 (1.96)		3.53 (1.29)	
		Northeast	3.86 (1.23)		3.02 (2.16)		3.54 (1.28)	
		South	3.66 (1.37)		2.95 (2.08)		3.48 (1.31)	
		West	3.67 (1.45)		3.3 (2.13)		3.6 (1.27)	

^a^N/A: not applicable.

^b^*P* values calculated using the t-test, the Mann-Whitney test (for 2 categories) or the Kruskal-Wallis test (for >2 categories).

^c^*P* values of <.05.

^d^Includes Asian, Pacific Islander, and other. Others excluded from test because of sparsity.

#### Problem Technology Use

In total, 62.5% (177/283) of the parents agreed with the statement “When my mobile electronic device alerts me to indicate new messages, I cannot resist checking them.” The problem technology use scale had high internal consistency, with a Cronbach α of .8 (Table 2). The mean level of perceived problematic mobile device use in general for parents in our sample was 3.72 (SD 2.07) on a 6-point scale where a higher score was more problematic (Table 2).

#### Parenting Technoference

Around 75.6% (214/283) of the parents reported that smartphones interfered in their parent-child interactions at least once daily. Parents in our sample reported a mean of 3.03 devices (SD 2.07) interfering in their interactions with their child on a daily basis (Table 2).

#### Sociodemographic Factors

Age, sex, race/ethnicity, and language in the home were all significantly associated with at least one of the primary measures (Table 2). In particular, younger parents perceived that they had a greater level of problem technology use in general and parenting technoference in particular and were more accepting of a technology-based tool to help reduce these problems. Males were slightly more accepting of technology-based solutions to reduce parenting technoference. Parents identifying as Black reported higher levels of problem technology use in general, whereas those identifying as Hispanic reported higher levels of parenting technoference*.* Parents who reported that they speak a language other than English (overwhelmingly Spanish) at home had higher levels of perceived parenting technoference. There was no significant association between parents’ reported technology use and their educational attainment, annual income, or geographic region (Table 2).

### Multiple Regression and Mediation Analyses

**Figure 1:**
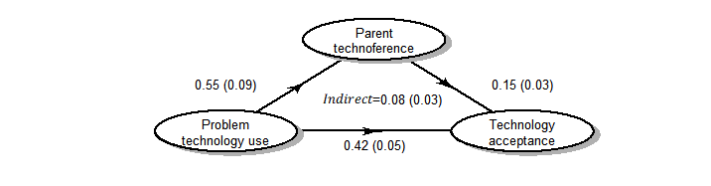
Results of the mediation analysis. Mean effect sizes and SDs are shown.

Controlling for sociodemographic factors, the association between parents’ acceptance of technology-based tools to combat technoference in parent-child interactions and their perceptions of their own problem technology use was high, with a large Cohen *d* effect size [41] of 0.98 (*P*<.001) and an η^2^ value of 27% of the total variance explained (Table 3). The effect size for the association between technology acceptance and parenting technoference was 0.51 (*P*<.001), with an η^2^ value of 6% of the variance explained (Table 3). None of the sociodemographic factors measured were significantly associated with technology acceptance in the regression models, once parents’ own perceptions of their problem technology use and parenting technoference were accounted for. The results of mediation analyses (Figure 1) indicate that the relationship between problem technology use and technology acceptance did not have a strong, indirect component that was mediated through the more specific parent technoference construct. The magnitude of the indirect effect was only 0.08, compared with 0.42 for the direct effect of problem technology use on technology acceptance, with 16% of the total effect mediated through technoference.

**Table 3:** Results of the final reduced regression model with technology acceptance as the outcome.

Variable		Regression estimate		Cohen *d*		OR (95% CI)		*P* value		Partial η^2^ value (%)	
Problem technology use		0.42		0.98		N/A^a^		<.001^b^		27	
Parenting technoference		0.14		0.51		N/A		<.001^b^		6	
Age of parent (years)		−0.006		−0.09		N/A		.48		2	
**Sex**								.18		2	
	Female		N/A		N/A		N/A				
	Male		0.22				1.24 (0.9, 1.71)				
**Education**								.30		3	
	Less than college degree		N/A		N/A		N/A				
	College degree		0.15		N/A		1.16 (0.87, 1.54)				
**Income (US $)**								.33		2	
	<25,000		N/A		N/A		N/A				
	25,000-<50,000		−0.11		N/A		0.9 (0.61, 1.32)				
	50,000-<75,000		−0.03		N/A		0.97 (0.65, 1.45)				
	75,000-<100,000		0.3		N/A		1.35 (0.88, 2.07)				
	>100,000		−0.03		N/A		0.97 (0.60, 1.56)				

^a^N/A: not applicable.

^a^*P*<.05.

## Discussion

In this national survey of parents of young children, we found high levels of acceptance for AI-based coaching tools designed to help reduce threats from parenting technoference to the nurturing environments needed in early childhood to ensure a healthy developmental trajectory to adulthood. This acceptance was most strongly related in regression models to 2 factors: perceived problematic parent technology use in general and perceived level of technoference in the parent-child relationship in particular. In comparison, none of the sociodemographic factors we examined explained a statistically significant portion of the variance in the acceptance of AI-based coaching tools once the factors measuring perceptions of their own susceptibility to the problem of technoference were considered.

Although we found no studies to date on the acceptability of AI-based parenting tools to compare with our results, our findings are somewhat consistent with those of related literature on the acceptability of AI tools in general and for technology-based tools in health care in particular. For example, in one recent study of factors influencing the use of in-home voice assistants, only household size, but not age and gender, influenced usage. In a study of physical activity–monitoring wearable devices, females were found to be slightly more accepting (higher PU) than males [42]. A review of technology-based parenting interventions concluded that although younger parents may be more open to parenting interventions, they may appeal to a wide range of income levels [14].

Although we were unable to find published studies reporting on the direct relationship between parenting technoference and age, gender, race, and income, the finding that younger parents had higher perceived levels of problem technology use and parenting technoference is consistent with the findings from studies on the relationship between smartphone addiction and age [43,44]. Given that technoference and problem technology use are higher in younger parents, whom we also found to be more open to AI-based tools to mitigate technoference, we suggest that young parents should be the focus of future research and development in this area. However, the lack of a significant association between perceived device use and acceptability and education or income level suggests that the ubiquity of mobile devices is making parenting technoference a broad public health problem requiring scalable solutions.

The finding that parents’ perceptions of their own technoference and problematic technology use explained by far the largest proportion of variance in acceptability of AI-based tools to reduce parenting technoference may reflect the robustness of the HBM, in which our technoference measure corresponds to parents’ perceptions of their own susceptibility to this problem [32]. Thus, helping parents increase their awareness of their own technoference should be considered as a potential lever for increasing the acceptability of scalable AI-based interventions to mitigate this threat to early childhood development.

Finally, the finding from regression and mediation analyses that the most important factor influencing acceptability of AI tools to reduce technoference was parents’ perceptions of general technology overuse compared with their perceptions of how much their technology interfered with their parenting warrants further investigation. AI-based coaching tools or any technology-augmented tools to help reduce parent technoference might be able to focus simply on reducing parents’ overall problematic technology use. In addition, further research is needed to uncover other characteristics of parents who view themselves as having problematic digital device use, given that we did not identify strong relationships between the sociodemographic factors we examined and the parent problem technology use construct.

The levels of perceived parent problematic use of technology and perceived amount by which technology interfered with daily parent-child interactions were higher in this study than in the first study reporting on these measures. In particular, in our study, parents reported an average of 3.03 (SD 2.07) devices interfering daily, compared with 2 devices in a previous study [9]. This is likely because the measurements in that study were taken between 2014 and 2016; secular trends toward increasing awareness of problematic technology use in general and technology interference in parenting in particular have occurred since then.

### Limitations

The limitations of this study include those commonly associated with web-based surveys. Parents responding to web-based surveys may be more accepting of technological interventions than parents who do not respond to web-based surveys, resulting in selection bias. Furthermore, although social desirability bias may have resulted in a desire to downplay one’s technology use during active parenting, previous research suggests that web-based surveys are no more susceptible to this type of bias than human-administered surveys [45].

Another limitation is that our sample had far more females (222/280, 79.2%) than are representative of the US population. This could have been because the survey focused on parenting topics. Our sample was not representative of the US population in other ways. Compared with the 2018 American Community Survey, the study’s respondents reported slightly lower income levels and slightly higher education levels; fewer spoke a language other than English at home, and fewer were ethnic minority respondents [46]. In addition, we were not able to assess the degree of bias that resulted from parents who were approached but elected not to participate and the observations that had to be dropped because of data quality criteria.

Finally, the interpretation of some scales is limited by a lack of established national standards. For example, no guidelines exist for determining a sufficiently high level of technology acceptance using the TAM-PU scale.

### Conclusions

AI-based tools may be acceptable to use as coaching aids to help a wide sociodemographic range of parents improve their attentiveness while caring for their young children, especially in the face of technoference from their own use of mobile devices. Designers and developmental specialists should work together to develop and test AI-based tools to reduce parenting technoference, with an initial focus on younger parents. Future investigations should validate whether it is sufficient to focus AI-based parenting supports to combat technoference on parents’ general overuse of digital technology rather than their specific problems with technoference and to identify other factors that influence the acceptability and utility of these supports.

## Appendix

Multimedia Appendix 1 Full questionnaire/survey.

## Abbreviations

AI: artificial intelligence
HBM: Health Belief Model
LENA: Language ENvironment Analysis
PU: perceived usefulness
TAM: technology acceptance model
