# Supplementary material for: Parents’ Perspectives on Using Artificial Intelligence to Reduce Technology Interference During Early Childhood: Cross-sectional Online Survey
Source: J Med Internet Res. 2021 Mar 15;23(3):e19461. doi: 10.2196/19461 (PMC8074848; doi:10.2196/19461)
Supplement: Multimedia Appendix 1 [file jmir_v23i3e19461_app1.docx]

TurkPrime Survey of Parent Perceptions of Technoference and AI Parenting Supports

Final Qualtrics Download of Questions and Codes

Start of Block: Consent Form

**Consent Form**
 
**DESCRIPTION:**  The reason we are doing this research study is to learn the best way to help parents ensure technology helps rather than interferes with their parenting practices. You are being asked to be part of this study because you have a child between the ages of 0 and 5. If you agree to be part of the study, we will ask you to fill out a survey with questions about your experiences parenting your young child. You also will be asked questions about the role of technology in your life. Finally, we will ask you some questions about your child, yourself, and your family.

**RISKS AND BENEFITS:** There are no risks to your health from being in this study. You may feel uncomfortable with some of the questions we ask you. You do not have to answer any questions that you feel uncomfortable with. We do not think there will be a direct benefit to you or your family from being in the study. We hope that what we learn will help other parents like you in the future. We cannot and do not guarantee or promise that you will receive any benefits from this study

**TIME INVOLVEMENT:** Today, we will ask you questions using a survey.  This will take about 10-15 minutes.
**PAYMENTS:** TurkPrime will provide you compensation for completing this important research task.
**PARTICIPANT’S RIGHTS:** If you have read this form and decide to be a part of this study, please understand that your being part of the study is voluntary. You are free to choose not to be a part of the study.  You have the right to stop being part of the study at any time without penalty or loss of benefits to which you are otherwise entitled. You have the right to not answer any questions you are not comfortable with. Your information will be kept private in all published and written information that comes from the study.
**Contact Information:**  Questions, Concerns, or Complaints: If you have any questions, concerns or complaints about this research study, its procedures, risks and benefits, you should ask the Project Director, Lee M Sanders. You may contact him at (650) 736-8567.

Injury Notification: If you feel you have been hurt by being a part of this study, please contact the Project Director, Lee Sanders at (650) 736-8567.

Independent Contact: If you are not happy with how this study is being done, or if you have any concerns, complaints, or general questions about the research or your rights as someone who is part of the study, please contact the Stanford Institutional Review Board (IRB) to speak to someone who is not on the research team at (650) 723-5244 or toll free at 1-866-680-2906.  You can also write to the Stanford IRB, Stanford University, MC 5579, Palo Alto, CA 94304.   The extra copy of this consent form is for you to keep.

- I consent, begin the study (1)
- I do not consent, I do not wish to participate (2)

Skip To: End of Survey If Consent Form = I do not consent, I do not wish to participate

End of Block: Consent Form

Start of Block: AI Survey Block 1

**TurkPrime Survey of Parent Perceptions of Technoference and Artificial Intelligence (AI)-based Parenting Supports**

**The aim of this survey is to understand how you think of your role as a parent to your child between the ages of 0 and 5. You are the expert.  We want to understand your honest opinions.  No identifying information about you (for example, your name) will be collected and shared for this survey.**

**Thank you!**

**First, we are interested in understanding the different goals or priorities that you think parents might have in raising their children. Please give your honest response to each item, using the following scale:**

| **How important is it for a parent to teach their child to...** | Not at all important (1) | Slightly important (2) | Important (3) | Very Important (4) | Extremely Important (5) |
| --- | --- | --- | --- | --- | --- |
| 1.Language skills (e.g., the names of objects, how to put together sentences, or to use proper grammar) (1) |  |  |  |  |  |
| 2.Math skills (e.g., the names of numbers, how to count, or how to add/subtract) (2) |  |  |  |  |  |
| 3.Motor skills (e.g., how to crawl, run or walk, how to feed themselves, or fine motor skills like using a pencil) (3) |  |  |  |  |  |
| 4.Emotional intelligence (e.g., how to seek help when they are upset, how to express their emotions, or how to calm them self down) (4) |  |  |  |  |  |
| 5.Independence (e.g., developing self-confidence, the ability to play on their own without an adult, or how to figure out how things work by themselves) (5) |  |  |  |  |  |
| 6. Social skills (e.g., the importance of following the rules, how to share and take turns, or respecting other people’s boundaries) (6) |  |  |  |  |  |

End of Block: AI Survey Block 1

Start of Block: AI Survey Block 2

**The following questions ask about your use of mobile electronic devices. By mobile electronic devices, we mean mobile phones and other devices -- including tablets (e.g., iPad, Kindle Fire, Galaxy Touch) and other handheld devices (e.g., iPod touch, AppleWatch, Kindle, Nintendo Switch).**

**In a typical day, for about how many HOURS do you use each of the following:**

|  | 0 hours (1) | <1 hour (2) | 1 hour (3) | 2 hours (4) | 3 hours (5) | 4+ hours (6) |
| --- | --- | --- | --- | --- | --- | --- |
| 7. A television (TV)` (1) |  |  |  |  |  |  |
| 8. A desktop or laptop computer (2) |  |  |  |  |  |  |
| 9. A mobile phone without internet connectivity (3) |  |  |  |  |  |  |
| 10. A mobile phone with internet connectivity (smartphone) (4) |  |  |  |  |  |  |
| 11. A tablet (e.g., iPad, Kindle Fire, Galaxy Touch) (5) |  |  |  |  |  |  |
| 12. Other handheld electronic devices (e.g., iPod touch, Apple Watch, Kindle) (6) |  |  |  |  |  |  |
| 13. A video game console (7) |  |  |  |  |  |  |

**In a typical day, for about how many HOURS do you use mobile electronic devices to do each of the following:**

|  | 0 hours (1) | <1 hour (2) | 1 hour (3) | 2 hours (4) | 3 hours (5) | 4+ hours (6) |
| --- | --- | --- | --- | --- | --- | --- |
| 14.Shopping (1) |  |  |  |  |  |  |
| 15. Playing games (2) |  |  |  |  |  |  |
| 16.Watching videos (3) |  |  |  |  |  |  |
| 17. Seeking general information (4) |  |  |  |  |  |  |
| 18. Seek information on parenting or caregiving (5) |  |  |  |  |  |  |
| 19. Seek information on your child's health (6) |  |  |  |  |  |  |
| 20. Post information on parenting or caregiving -- to help others (7) |  |  |  |  |  |  |
| 21. Post information on child health -- to help others (8) |  |  |  |  |  |  |

**In a typical day, how many TIMES does each of the following devices interrupt a conversation or activity between you and your child?**

|  | 0 times (1) | 1 time (2) | 2 times (3) | 3 times (4) | 4+ times (5) |
| --- | --- | --- | --- | --- | --- |
| 22. A television (TV) (1) |  |  |  |  |  |
| 23. A desktop or laptop computer (2) |  |  |  |  |  |
| 24. A mobile phone without internet connectivity (3) |  |  |  |  |  |
| 25. A mobile phone with internet connectivity (smartphone) (4) |  |  |  |  |  |
| 26. A tablet (e.g., iPad, Kindle Fire, Galaxy touch) (5) |  |  |  |  |  |
| 27. Other handheld device (e.g., iPod touch, Apple Watch, Kindle) (6) |  |  |  |  |  |
| 28. A video game console (7) |  |  |  |  |  |

End of Block: AI Survey Block 2

Start of Block: AI Survey Block 3

| **Please rate your agreement with each statement, using the following scale:** | Strongly Disagree (57) | Disagree (58) | Slightly Disagree (59) | Slightly Agree (60) | Agree (61) | Strongly Agree (62) |
| --- | --- | --- | --- | --- | --- | --- |
| 29.When my mobile electronic device alerts me to indicate new messages, I cannot resist checking them. (5) |  |  |  |  |  |  |
| 30.I often think about calls or messages I might receive on my mobile phone. (6) |  |  |  |  |  |  |
| 31.I feel like I use my mobile phone too much. (7) |  |  |  |  |  |  |
| 32.Mobile electronic devices make parenting easier. (8) |  |  |  |  |  |  |
| 33.I am worried about the impact of my mobile electronic device use on my child. (9) |  |  |  |  |  |  |
| 34.I would like help in limiting my mobile electronic device use around my child. (10) |  |  |  |  |  |  |
| 35.Mobile electronic devices make parenting harder. (11) |  |  |  |  |  |  |
| 36.I have tried to limit my mobile electronic device use around my child. (12) |  |  |  |  |  |  |
| 37.My mobile electronic device use sometimes makes my child feel like I’m not paying attention to her or him. (13) |  |  |  |  |  |  |

| **Please rate your agreement with each statement, using the following scale:** | Strongly Disagree (48) | Disagree (49) | Slightly Disagree (50) | Slightly Agree (51) | Agree (52) | Strongly Agree (53) |
| --- | --- | --- | --- | --- | --- | --- |
| 38. I would like to use electronic devices more effectively, when in my child’s presence. (1) |  |  |  |  |  |  |
| 39. To help me use electronic devices effectively in my child’s presence - I would **attend a class.** (2) |  |  |  |  |  |  |
| 40. To help me use electronic devices effectively in my child’s presence - I would **attend a support group with other parents.** (3) |  |  |  |  |  |  |
| 41. To help me use electronic devices effectively in my child’s presence - I would consider **help from a coach in my home.** (4) |  |  |  |  |  |  |

End of Block: AI Survey Block 3

Start of Block: AI Survey Block 4

**Some electronic devices are currently being designed to HELP you have a better connection with your child – by coaching you or giving you meaningful, real-time feedback.**   **Imagine such a “computer-assisted coach,” which you could use in your home to get feedback on your use of electronic devices while caring for your child.  The computer-assisted coach would automatically analyze computer vision and other data to provide the feedback. Whenever you want, you could turn this computer-assisted coach on or off.**

| **Please rate your agreement with each statement below, using the following scale:** | Strongly Disagree (43) | Disagree (44) | Slightly Disagree (45) | Slightly Agree (46) | Agree (47) | Strongly Agree (48) |
| --- | --- | --- | --- | --- | --- | --- |
| 42. Using a computer-assisted coach while caring for my child would quickly **feel like a normal part of life**. (35) |  |  |  |  |  |  |
| 43. Using a computer-assisted coach while caring for my child would **help me be more aware of my device use around my child.** (36) |  |  |  |  |  |  |
| 44. Using a computer-assisted coach while caring for my child would **improve my interactions with my child.** (37) |  |  |  |  |  |  |
| 45. Using a computer-assisted coach while caring for my child would **help me be a better parent.** (38) |  |  |  |  |  |  |
| 46. Using a computer-assisted coach while caring for my child would **feel like “too much” technology.** (39) |  |  |  |  |  |  |
| 47. Using a computer-assisted coach while caring for my child would **raise privacy concerns.** (40) |  |  |  |  |  |  |

| **Please rate your agreement with each statement below, using the following scale:** | Strongly Disagree (44) | Disagree (45) | Slightly Disagree (46) | Slightly Agree (47) | Agree (48) | Strongly Agree (49) |
| --- | --- | --- | --- | --- | --- | --- |
| 48. Using a computer-assisted coach while caring for my child would **help me notice more quickly when my device use is interfering with my caregiving.** (19) |  |  |  |  |  |  |
| 49. Using a computer-assisted coach while caring for my child would **help me keep my attention focused on my child.** (20) |  |  |  |  |  |  |
| 50. Using a computer-assisted coach while caring for my child would **help me discuss issues with my child’s doctor.** (21) |  |  |  |  |  |  |
| 51. Using a computer-assisted coach while caring for my child would **help me discuss issues with my child’s teacher.** (22) |  |  |  |  |  |  |
| 52. Using a computer-assisted coach while caring for my child would **help me discuss issues with my child's other parent or other caregiver.** (23) |  |  |  |  |  |  |
| 53. Using a computer-assisted coach while caring for my child would **be useful to me.** (24) |  |  |  |  |  |  |

End of Block: AI Survey Block 4

Start of Block: AI Survey Block 6

**Finally, some brief questions about you and your family.**

Q12 54. What is your age?

▼ 18 (137) ... 100 (219)

Q14 55. What is your gender?

- Male (1)
- Female (2)
- Other (4) ________________________________________________

Q15 56. Do you consider yourself to be Hispanic or Latino/a?

- Yes (1)
- No (2)

Q16 57. What race do you consider yourself to be?

- White or caucasian (2)
- Black or African American (3)
- Hispanic or Latino (4)
- Asian (5)
- Native Hawaiian or Pacific Islander (6)
- American Indian or Alaska Native (7)
- Other (8) ________________________________________________

Q17 58. How well do you speak English? Would you say...?

- Very well (1)
- Well (2)
- Not well (3)
- Not at all (4)

Q18 59. What language do you usually speak at home?

- English (1)
- Spanish (2)
- Other (3) ________________________________________________

Q19 60. How long have you lived in the United States?

- I was born in the U.S. (1)
- Less than 1 year (2)
- 1-2 years (3)
- 2-5 years (4)
- More than 5 years (5)

Q20 61. How would you describe your marital status?

- Single, never married (1)
- Living with a partner (2)
- Married (3)
- Separated (4)
- Divorced (5)
- Widowed (6)
- Other (7) ________________________________________________

End of Block: AI Survey Block 6

Start of Block: AI Survey Block 7

Q21 62. Including yourself, how many people live in your home?

▼ 1 (71) ... 11+ (81)

Q22 63. How many children do you have?

▼ 1 (7) ... 11+ (17)

Q23 64. What is the age of your YOUNGEST CHILD?

▼ 1 (7) ... 11+ (17)

Q24 65. What is the highest level of school you have completed or the highest degree you have received?

- Less than high school degree (1)
- High school degree or equivalent (e.g., GED) (2)
- Some college but no degree (3)
- Associate degree (4)
- Bachelor degree (5)
- Graduate degree (6)

Q25 66. Right now, do you work?

- Yes (1)
- No (4)

Skip To: Q27 If 66. Right now, do you work? = No

Q26 67. Are you working part time or full time?

- Part-time (1)
- Full-time (2)
- Other (3) ________________________________________________

Q27 68. Please mark the group that is closest to your total household income in the past 12 months.

- Less than $25,000 (1)
- Between $25,000 and $49,999 (2)
- Between $50,000 and $74,999 (3)
- Between $75,000 and $99,999 (4)
- Between $100,000 and $124,999 (5)
- Between $125,000 and $149,999 (6)
- Between $150,000 and $174,999 (7)
- Between $175,000 and $199,999 (8)
- More than $200,000 (9)

End of Block: AI Survey Block 7

Start of Block: Thank you

Q28 **THANK YOU SO MUCH FOR YOUR PARTICIPATION! These answers will help us improve the health and well-being of children.**
 
**If you have any questions about this survey, please contact ParentingAI@stanford.edu**

End of Block: Thank you
